# Putting the Spring back into the Hare (*Pedetes capensis*): Meat Chemical Composition of an Underutilized Protein Source

**DOI:** 10.3390/foods9081096

**Published:** 2020-08-11

**Authors:** Sara Wilhelmina Erasmus, Louwrens Christiaan Hoffman

**Affiliations:** 1 Food Quality and Design Group, Wageningen University and Research, P.O. Box 17, 6700 AA Wageningen, The Netherlands; sara.erasmus@wur.nl; 2 Department of Animal Sciences, Faculty of Agriculture and Forestry Sciences, University of Stellenbosch, Private Bag X1, Matieland 7602, South Africa; 3 Centre for Nutrition and Food Sciences, Queensland Alliance for Agriculture and Food Innovation (QAAFI), The University of Queensland, Agricultural Mechanisation Building A. 8115. Office 110, Gatton 4343, Australia

**Keywords:** alternative proteins, springhare, underutilized animal-based foods

## Abstract

Alternative protein sources are gaining increasing global attention as a solution to address future protein demands. Determining the chemical composition of meat alternatives is vital to confirm that it is nutritious, but also to increase product value and promote its utilization. The carcass characteristics and chemical composition of springhare, an underutilized protein source, was found to be comparable to that of commercially reared rabbits. Hence, its introduction into the commercial supply chain would likely not offset consumers accustomed to purchasing rabbit/hare meat. Springhare meat had a high protein content (~22.5 g/100 g meat) and low lipid (<1.3 g/100 g meat) content. The meat’s fatty acids mainly comprised C18:2*n*6c (γ-linoleic acid; 24%), C18:0 (stearic acid; 20%), C16:0 (palmitic acid; 19%), C20:4*n*6 (arachidonic acid; 15%) and C18:1*n*9c (oleic acid; 13%). Although sex did not significantly influence the carcass characteristics and meat composition, season did have an effect (*p* < 0.05) on the fatty acid profile. The meat harvested in summer had higher (*p* < 0.05) concentrations of favorable unsaturated fatty acids, C18:2*n*6c, C18:3*n*6, C18:3*n*3 (α-linolenic acid), C20:2*n*6 (eicosadienoic acid), C20:3*n*3 (eicosatrienoic acid), compared to the meat obtained in winter, which contained more (*p* < 0.05) saturated fatty acids. The results verify that springhare can be utilized as a viable alternative protein source.

## 1. Introduction

In view of the increasing global population, agricultural practises will inevitably have to adapt to ensure food security, and specifically to produce protein that is accessible, affordable, healthy, and sustainable. Animal or muscle meat is very nutritious, being high in protein and containing all the essential amino acids together with vitamins and minerals. However, animal-based proteins are often criticized for their contribution towards global warming, while alternatives such as plant-based proteins, lab-grown meat, and insects are more frequently offered as the solution to feeding the masses in the future. Yet, one scenario which is often overlooked is the utilization of animal-based underutilized animal species and the harvesting of wild animals to control the population size as part of an effective and sustainable conservation strategy. The meat obtained through these practises can serve as valuable protein.

Hoffman [1] already emphasised the need to increase meat production through the utilization of naturally occurring game animals. These wild animals can either be hunted or commercially farmed and consumed as ‘bushmeat’ through trade in informal or rural markets, or as game meat or venison through formal meat trade. The above-mentioned review highlights the importance of the sustainable management and production of these unconventional meat sources, but it also points out the importance of generating scientifically accurate information on the yield and nutritional value (proximate and fatty acid composition) of the meat—especially to successfully market these species [1]. Various studies on the composition and quality of game meat, that emphasise its high protein and low fat contents, its optimal ratio of unsaturated to saturated fatty acids, and unique aroma and flavour have been performed [2,3,4,5].

It is important to note that there is a strong geographical link associated with non-traditional or unconventional meat sources, where a specific species will be familiar in some countries and underutilized in other countries. For instance, rabbit/hare meat is regularly consumed in Europe, certain North African countries, and China [6,7,8], while in southern African countries (for example, South Africa) it is less popular in the formal market but readily consumed by rural communities [9]. Culture, religion, beliefs, age, accessibility, and education all influence the popularity of a meat source and the consumer’s willingness to purchase the product [8]. However, by informing consumers about the beneficial nutritional value, one could increase their appreciation for the product and act as a driver for its demand [10]. The latter is crucial for the establishment of an economically sustainable supply chain for alternative or underutilized meat sources.

**Figure 1:**
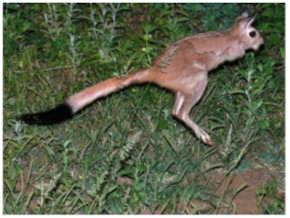
The South African springhare (*Pedetes capensis*) (photo by Bernard Dupont [12]; Shared according to CC BY-SA 2.0).

In South Africa, hunting is often performed by farmers on animal species that either cause damage to crops and/or compete with livestock for feed, are regarded as pests, or which prey on livestock. An animal species that is hunted as pests or as part of a recreational activity is the springhare (*Pedetes capensis*), depicted in Figure 1. Springhares are not hares nor rabbits, but the largest, hopping, nocturnal rodent species in southern Africa. They have a sandy, cinnamon-coloured skin, with a long fluffy tail that ends in a dark brown or black coloured tip. Springhares are viewed as abundant, especially in the Northern parts of the country where there are enough sandy areas for burrowing, and open short grassland for foraging [11]. It is roughly estimated that there are between 2 to 11 million springhares in South Africa, of which 2 to 8 million (74% of the population) would be mature adults. The species is listed as ‘Least Concern’ on the Global Red List, making it a suitable species, if conserved appropriately, to be used as an alternative meat source [11].

Springhare is recognized to be a century-old meat source for the indigenous Bushmen (San people) of southern Africa [13]. They are one of the 17 species that are systematically hunted by the San and form an important part of their diet. In rural communities, springhare are known to be an important source of protein. It has been previously estimated that in 1973 [14], 2.5 million springhares were consumed annually in Botswana, while in 1991, at least 3.3 million kilograms of the meat (equal to 30,000 cattle) reached the Botswana market as bushmeat [15]. Although these studies are dated and similar studies have not yet been performed in South Africa, it is evident that springhare can make a significant contribution to the protein consumption in both informal and commercial markets.

Peinke et al. [11] recently suggested that the research priorities for springhares should focus on the economic value of the species together with sustainable harvesting approaches. For the food industry, this would mean the economic value as a protein source. For its introduction into the food, chain it is important to determine the chemical composition of the meat in addition to the carcass characteristics as this is also connected to the quality of the meat. The aim of this study was, therefore, to determine the carcass characteristics and chemical composition of commercial meat cuts (loins and hind legs) of female and male springhare harvested during summer and winter. This study is the first of its kind that explores the potential for using springhare meat as an alternative protein source

## 2. Materials and Methods

### 2.1. Experimental Layout and Sample Collection

Springhares were harvested in Kimberley (Northern Cape Province, South Africa) during the summer (6 March) and winter (10 July) season (Ethical clearance number: SU-ACUM13-00010). Ten animals, five males, and five females were sourced per season on the same day. The animals were shot using a 12-gauge shotgun (Nr. 3 pellets) during the night, after being blinded with a spotlight, according to standard hunting procedures, after which they were transported to a game processing facility where they were weighed (dead weight) and dressed. The springhares were not bled as bleeding was seen to be internal due to the shotgun pellets; it was noted that the pellets had little/no impact on the meat yield. The dressing procedure steps were as follows: (1) Removal of the front paws; (2) Removal of the HL at the cross joint; (3) Removal of the head at the axis-atlas joint; (4) The skin was slit along the belly and the skin then pulled off with gentle tugs; (5) The belly was slit open and all the intestines removed for evisceration (not weighed); (6) After evisceration, the carcass was washed and weighed and left for 24 h in a refrigerator (4–7 °C,) before being vacuum-packed and frozen at −20 °C until further processing. The sex was easily distinguished with the males having large testicles inside the body. In total, 20 springhares were used for the study. The frozen carcasses were transported to the Meat Science laboratory at the Department of Animal Sciences (Stellenbosch University, Matieland, South Africa). They were then left to defrost overnight in a refrigerator at ~4 °C before the carcasses were dissected and the meat deboned. The data for the carcass characteristics, as shown in Table 1, were collected before and after slaughter and carcass dissection and deboning. Carcasses were dissected into the different retail cuts according to standard guidelines [16]. The left and right *Longissimus thoracis et lumborum* (LTL) muscles (from the intermediate part) as well as all the HL muscles (from the hind part) of the carcasses were removed and homogenised for chemical analyses. The LTL muscles were removed between the last thoracic and the first lumbar vertebra to the 6th and 7th lumbar vertebra. The HL muscles included *os coxae* and the posterior part of *m. iliopsoas*: *m. psoas major* and *m. iliacus* (*par lateralis* and *pars medialis*). The homogenised samples were then vacuum-packed and stored at −20 °C in the absence of light until the chemical analyses were conducted.

### 2.2. Chemical Compositional Analyses

The samples were removed from the −20 °C freezer to defrost overnight in a refrigerator at 4–7 °C before they were chemically analysed.

#### 2.2.1. Proximate Analysis

The proximate composition of the samples was determined in g per 100 g. The moisture and ash contents were determined according to AOAC official method 934.01 and 942.05, respectively [17]. The total protein content was determined with the Dumas method 992.15 [17]. A 0.15 g defatted, dried (at 60 °C for 24 h), and finely ground meat sample encapsulated in a Leco^TM^ foil sheet was analysed using a Leco nitrogen/protein analyser (FP-528, Leco Corporation). Ethylenediamine tetra acetate (Leco Corporation, part number 502-092, lot number 1055, 3000 Lakeview Avenue, St. Joseph, MO, USA, MI 49085-2396, USA) was used as the standard for the calibration of the nitrogen analyser throughout the analyses to ensure accuracy and recovery rate of each sample. The total lipid content was determined on a 5 g muscle sample using a chloroform/methanol (2:1, *v/v*) extraction [18].

#### 2.2.2. Fatty Acid Analysis

The fatty acids of the meat samples were extracted using a 2:1 (*v/v*) chloroform:methanol solution containing 0.01% butylated hydroxytoluene (BHT) (Sigma-Aldrich Inc., catalogue number B-1378, 3050 Spruce Street, St. Louis, MO 63103, USA) as antioxidant [19]. Heptadecanoic acid (C17:0) (Sigma-Aldrich Inc., catalogue number H3500, 3050 Spruce Street, St. Louis, MO 63103, USA) was used as an internal standard to quantify the individual fatty acids present in the meat. An aliquot of the extracted fatty acids was converted into corresponding methyl esters through transmethylation using a methanol:sulphuric acid (19:1; *v/v*) solution. The fatty acid methyl esters (FAMES) were determined by gas chromatography.

Homogenised meat samples, stored at −80 °C, were defrosted before determination of its long-chain fatty acid content (intramuscular). A 2 g meat sample was weighed off into an extraction tube (205 × 30 mm, Pyrex). This was followed with the addition of 20 mL 2:1 (*v/v*) chloroform:methanol solution and 500 µL internal standard. The meat sample together with the extraction solvent was homogenised for 10 s by means of a polytron mixer (Kinematica AG Homogenizer, PT-500 E, serial number PF-799-0004-02-19). The homogenised content was then transferred to an extraction funnel (0 porosity disc) covered with glass microfibre filter paper (Whatman, GF/A, 47 mm diameter, catalogue number 1820-047) leading into a 50 mL volumetric flask. A volume of 10 mL 2:1 (*v/v*) chloroform:methanol solution was added to the residue, the mixture homogenised with the polytron (to rinse), filtered and the final volume made up to 50 mL. A 250 μL aliquot of the filtered phase was transferred to a Klimax tube (with Teflon lined screw cap) and dried under nitrogen in a 45 °C scientific water-bath. Two mL of the transmethylating agent (19:1, *v/v*, methanol/sulphuric acid solution) was added to each sample and placed in a 70 °C waterbath for 2 h. Following transmethylation, the mixture was cooled to room temperature (21 °C) and the FAMES extracted with 1 mL distilled water and 2 mL hexane by transferring the top hexane phase to a clean Klimax tube and then drying it under nitrogen in a 45 °C waterbath. For chromatographic analysis, 100 µL hexane was added to the dried FAMES sample and transferred into an autosampler vial with 0.1 mL insert and closed with a PTFE/red silicone septa screw-cap (SupelcoTM, 595 North Harrison Rd, Bellefonte, PA 16823-0048, USA).

The FAMES were determined by gas chromatography in an Agilent 6890 model gas chromatograph (Agilent, Palo Alto, CA, USA), coupled with a flame-ionization detector and split injector port, set at 280 °C and 240 °C, respectively. Split injection was made at a 5:1 ratio, while the chromatographic separation of the FAMES was performed on a DB-225MS capillary column (30 m, 0.25 mm internal diameter, 0.25 µm film thickness, Agilent J&W GC Columns, part number 122-2932). Hydrogen (40 mL/min flow rate) was used as carrier gas and helium as makeup gas, with a combined flow of 30 mL/min. The oven was operated as follow: 50 °C for 1 min; ramped up to 175 °C at 25 °C/min and held for 0 min followed by ramping up to 210 °C at 2 °C/min and held for 6 min, then ramped up to 220 °C at 1 °C/min and held for 0 min followed by ramping up to 235 °C at 1.5 °C/min and held for 2 min. The sample volume injected was 1 µL and the run time approximately 45 min. Fatty acids were identified by comparing their retention times to those found in a standard FAME mixture (SupelcoTM, 37 Component FAME mix, C4-C24, 10 mg/mL in CH2Cl, catalogue number 47885-U, North Harrison Road, Bellefonte, PA 16823-0048, USA). The results were recorded as percentage (%) of the total FAMEs.

### 2.3. Statistical Analysis

The data were statistically analysed using SAS^®^ Enterprise Guide 7.1 statistical software (Statistical Analysis System, Version 7.15 HF3, 7.100.5.6132, 32-bit, 2017, SAS Institute Inc., Cary, NC, USA) and XLSTAT^®^ statistical software (Version 2019.3.2; Addinsoft, NY, USA; https://www.xlstat.com) for analysis of variance (ANOVA). Pre-processing of the data involved using the Shapiro-Wilk test to test for deviation from normality [20]. In one case where the deviation from normality was significant (*p* ≤ 0.05), an outlier in the data was identified for one variable (total lipid: one summer, female HL sample) and removed. After confirming that the data was symmetrically distributed, one-way ANOVA was carried out. In cases where the data did not meet the conditions for parametric tests, the Kruskal-Wallis non-parametric test together with Dunn’s procedure for multiple pairwise comparisons were performed. Fisher’s Least Significant Differences (LSD) were calculated at a 5% significance level to compare variable means. A probability level of 5% was considered significant. Multivariate statistical techniques were used to find significant patterns and associations in the collected data with principal component analysis (PCA) being used to visualise sample grouping and associations, while correlations were determined by means of the Pearson’s correlation coefficient (*r*) [21].

## 3. Results and Discussion

### 3.1. Carcass Characteristics

Although the springhare can be mistaken for rabbits and hares due to their movement and phenotypic traits. They are taxonomically more closely related to rodents, as the species (*Pedetes capensis*) falls under the genus *Pedetes* (order of *Rodentia)*, where phylogenetic analyses support the grouping of the *Pedetidae* family with *Sciurognathous* (rat-like) characteristics [11]. In this study, the chemical composition of the meat will mostly be compared to that of rodents as well as rabbits/hare; given that there have been numerous studies performed on the latter and the similarities in the conformation of the animals.

Considering the dead (slaughter) and carcass weights of the springhare in Table 1, the average weights ranged from 2.4–2.6 kg and 1.5–1.6 kg, respectively, with season and sex not having a significant influence on the weights. As springhares breed year-round, breeding breaks or associated changes in body weight due to pregnancy or changes in body condition were not apparent. The weights were the same as those reported for New Zealand and Phendula meat rabbits [22]. Mature springhares typically weigh 3–4 kg [23,24], which are at the higher end of the dead weights recorded for the springhare of this study (Table 1). Dress out percentage and the weight of the tails were the only two characteristics that differed significantly due to the effect of season. Springhare carcasses sourced during summer had a higher dress out percentage and tail weight compared to the carcasses from winter (*p* < 0.05). The increase in tail weight during summer could be linked to the activity or the diet of the animal.

The springhare uses the posterior part of the body to move by jumping on its hind legs and feet (ricochetal locomotion), while the tail supports the movement. During the summer months, the springhare could have been more active due to the increase in temperature during the night as springhare are nocturnal and prevalence of natural predators. The increase in activity will likely enhance muscle development in the posterior part of the animal. However, contrary to the assumption of increased activity during summer at higher temperatures is the fact that low temperatures have very little effect on springhare activity [24]. Therefore, diet could also have influenced the weight of the springhare, as it was noted that the grass was very sparse during the winter harvest in Kimberly (July) as a drought was being experienced. Consequently, the springhare would have consumed less due to the limited availability of natural plants.

Although not significantly different, the intact HL of the summer springhare had slightly higher values compared to the HL of the winter springhare (~646 g vs. ~617 g, respectively). The HL also weighed roughly 100 g more than that previously reported for hare meat [25]. Similarly, the male springhare showed slightly higher values for their forearms (~98 g vs. ~91 g), LTL (~227 g vs. ~195 g), HL (~650 g vs. ~614 g) and tails (~67 g vs. ~65 g) compared to the female springhare; a trend to note in future studies. Similar results were seen for meat rabbits where the male animals tended to have heavier weights for the different cuts compared to females [22]. Furthermore, it is possible that the lack of sex effect could be linked to age, but due to sampling bias, it was not possible to determine the age of the animals during the night cull.

### 3.2. Chemical Composition

#### 3.2.1. Proximate Composition

The results for the proximate composition (g/100 g meat) of the different springhare muscles are shown in Table 2. Following the analysis of variance (ANOVA) on season and sex, respectively, the only significant effect was found for season, where the ash content of both muscles differed in addition to the moisture content of the HL. However, given that proximate analyses generally have a higher degree of error and that the values are relatively close, no solid conclusions can be made about the effect of season (if any) on the proximate composition of the meat. Yet, it is important to note that the meat has a favourable high protein content and low total lipid content; it is noteworthy to mention that none of the animals used in the study had any subcutaneous fat or deposits of mesenteric fat and/or kidney fat. When compared to the general composition of rabbit and hare meat [6,26], springhare meat had a similar moisture, protein, and ash content. Dalle Zotte and Szendrő [6] reported a slightly higher lipid content for the LTL (1.8 ± 1.5 g/100 g) and HL (3.4 ± 1.1 g/100 g) of rabbit meat, while Króliczewska et al. [26] also reported higher crude fat contents (2.75–3.47 g/100 g) for hare and rabbit meat.

#### 3.2.2. Fatty Acid Composition of the Springhare Meat

The overall fatty acid composition (% total FAME) of the springhare meat (LTL and HL combined) is shown in Table 3. The following fatty acids were the most abundant (constituting 91%): C18:2*n*6c (24%); C18:0 (20%); C16:0 (19%); C20:4*n*6 (15%); C18:1*n*9c (13%). The results are similar to that from rabbits and hare [26,27] and rodents [28], except for the C18:0 and C18:1*n*9c which tended to be roughly 10% higher and lower, respectively. Overall, the springhare meat contained a high concentration (46%) of total polyunsaturated fatty acids (ΣPUFA), of which the total *n*-6 PUFA (Σ*n*-6) and *n*-3 PUFA (Σ*n*-3) were 40% and 6%, respectively. The ratio of PUFA:SFA and *n*-6:*n*-3 were 1 and 7, respectively. For a healthy diet, the recommended values for PUFA:SFA are 0.45 or above, and 4.0 or below for *n*-6:*n*-3 fatty acids ratios [29]. Hence, the springhare meat has a satisfactory PUFA:SFA ratio, but an unfavourable *n*-6:*n*-3 ratio. A high dietary *n*-6:*n*-3 fatty acids ratio may promote the pathogenesis of many diseases, including cardiovascular disease, cancer, and inflammatory and autoimmune diseases [30]. The high content of *n*-6 fatty acids are mainly due to the concentration of linoleic acid and arachidonic acid in the meat. Króliczewska et al. [26] found similar high *n*-6 concentrations and *n*-6:*n*-3 fatty acids ratios for hare and rabbit meat. However, the authors concluded that since the hare meat had a higher ΣPUFA (40–46%) than rabbit meat (27–29%), the atherogenic index was significantly lower for hare meat, which suggests that the alternative consumption of hare meat may help to reduce cardiovascular diseases. For this reason, the same assumption can be made for springhare meat. Another important difference to note between species is the total saturated fatty acids (ΣSFA) and mono-unsaturated fatty acids (ΣMUFA); springhare tend to have about 5% higher concentrations for ΣSFA (40%) and 10% lower concentrations for ΣMUFA (14%), respectively, when compared to the meat of hares (34–35% and 18–23%), rabbits (37–38% and 29–34%), capybara (39% and 28%), and guinea pig (33% and 26%) [26,28].

#### 3.2.3. Effect of Season on the Fatty Acid Composition

The results for the fatty acid composition (% total FAME) of the different springhare muscles based on season are shown in Table 4; various significant differences were determined for the mean values of the fatty acids using the Kruskal-Wallis test. The fatty acids of the HL meat show a trend with compositional differences in meat from summer and winter. When compared to the summer HL meat, the winter HL meat showed somewhat higher mean values for ΣSFA (41% vs. 38%). Accordingly, there is a propensity for the winter HL meat of the springhare to contain a slightly higher content of saturated fatty acids compared to the summer meat. Two SFA that are exceptions are C20:0 (arachidic acid) and C21:0 (heneicosylic acid) as the summer HL meat contained higher concentrations of these SFA when compared to the winter meat. The springhare harvested in summer, specifically the HL, had the highest mean values, significantly more than that of the winter meat, for the following unsaturated fatty acids: C17:1 (0.36 ± 0.048%); C18:3*n*6 (0.38 ± 0.053%); C18:3*n*3 (2.30 ± 0.512%); C20:2*n*6 (0.07 ± 0.010%); C20:3*n*3 (0.34 ± 0.042%); PUFA:SFA (1.27 ± 0.062); Σ*n*-3 (6.93 ± 0.629%).

**Figure 2:**
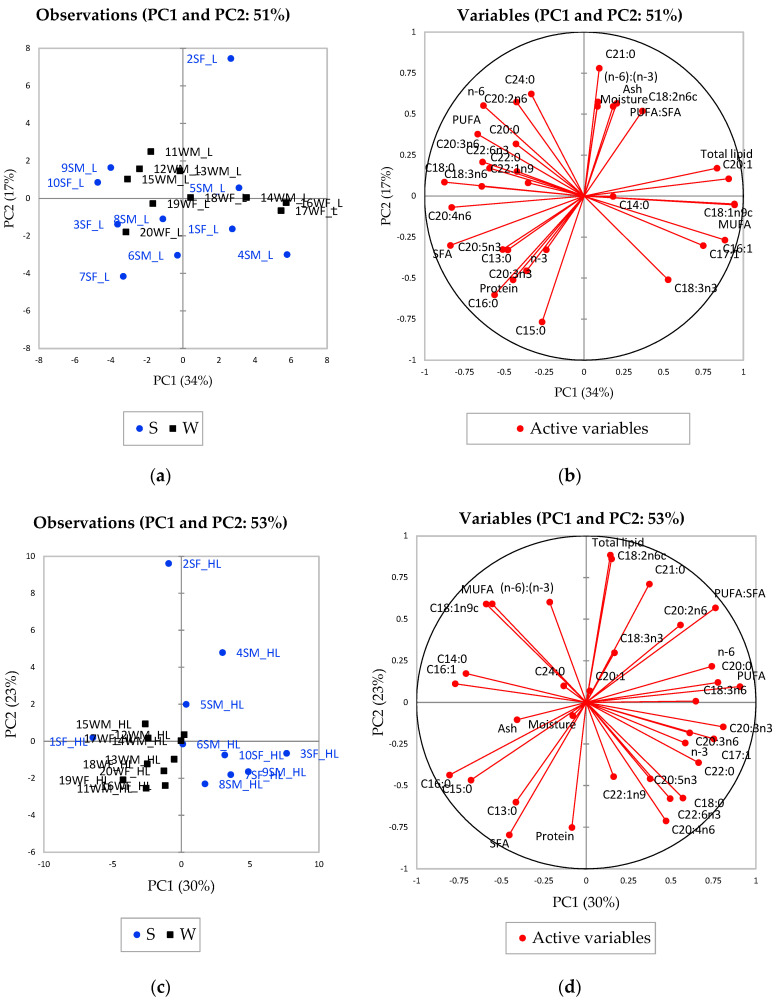
The principal component analysis (PCA) observations plots (**a**,**c**) and variables plots (**b**,**d**) for the chemical composition (moisture, protein, total lipid, ash and fatty acids) of the different male (M) and female (F) springhare loin (L) (PCA plots **a**,**b**) and hind leg (HL) (PCA plots **c**,**d**) meat samples sourced during summer (S) and winter (W). Example of sample code: (1SF_HL) number 1, summer, female, hind leg. For interpretation of the colours, refer to the electronic version of the article.

A principal component analysis (PCA) was performed to explore and visualise the meat sample groupings based on its chemical composition according to season. The results for the LTL and HL samples are shown in Figure 2. Figure 2c,d shows the grouping of the summer HL samples on the right side of the plot vs. the winter HL samples on the left side along PC1 (which explains 30% of the variation). For the LTL samples, a similar trend was seen although less apparent and with more overlap between summer and winter samples (Figure 2a,b). This separation in grouping is due to the higher content of saturated fatty acids in the winter meat samples, and unsaturated fatty acids in the summer meat samples (Table 4).

The highest mean value for ΣSFA was found for the winter HL springhare meat (Table 4), together with a high association on the left lower side of the variables plots (Figure 2d) along PC1. ΣSFA correlate positively with C13:0 (*r* = 0.742; *p* = 0.0002), C15:0 (*r* = 0.717; *p* = 0.0004), C16:0 (*r* = 0.781; *p* < 0.0001), and negatively with total lipid (*r* = −0.724; *p* = 0.0003) and PUFA:SFA (*r* = −0.876; *p* < 0.0001). The winter HL springhare meat also had the lowest PUFA:SFA (Table 4) and total lipid (Table 2) means. Hence, it is expected that for winter springhare HL meat an increase in saturated fatty acids will coincide with a decrease in total lipid content and PUFA:SFA (together with PUFA and *n*-6). The opposite trend is evident for summer HL meat as it contained the highest (*p* < 0.05) content of C20:3*n*3 (correlates with PUFA: *r* = 0.730; *p* = 0.0003), C18:3*n*6 (correlates with C20: *r* = 0.711; *p* = 0.0004; C17:1: *r* = 0.824; *p* < 0.0001), and C17:1 (correlates with: *r* = 0.724; *p* = 0.0003), together with a high association on the right lower side of the variables plots (Figure 2d) along PC1. These variables can be regraded are some of the main drivers for the separate grouping of summer and winter HL meat. Undoubtedly, the summer meat has a fatty acid profile with high concentrations of PUFA. To explain the variation in fatty acid profiles, it is important to consider the vegetation and consumptive behaviours of springhare.

Springhares are entirely herbivorous, selective grazers, and mainly eat green grass seeds which are high in protein and water, but sometimes they also feed on grass stems, leaves, corms, roots, and rhizomes [11,24]. They can also uproot entire plants to only eat a selected part, discarding the rest. Their diet can change depending on the season, which coincides with the planting and harvesting periods for crops, and the abundance of natural vegetation. This change in vegetation with the change in season can bring forth a change in the composition of the meat as it is well known that the fatty acid composition in, for example, rabbit meat, is directly influenced by diet [31]. Therefore, it is important to consider the environmental conditions when exploring the seasonal effects on the fatty acid composition of springhare meat.

The springhare used in this study were harvested during the summer and winter periods near Kimberley, a town situated in the Free State province of South Africa. This region consists of plains with summer rainfall and vegetation mainly consisting of Kimberley thornveld (Savanna biome) and to a lesser extent that of the Western Free State clay grassland (grassland biome) [32]. It was noted that during the winter harvest in Kimberly (July 2013), the grass was very sparse as a drought was being experienced. Therefore, less grassland vegetation was available to the springhare and it can thus be postulated that the springhare had to adapt by adjusting their diet from grasses (predominant during the summer) to commercial winter crops much as maize that are harvested during winter – the springhare were all harvested in areas in close proximity to maize (corn) fields that were on the point of harvest.

Grass is naturally high in α-linolenic acid [33], while grain-based diets and diets containing other seeds and plants are high in linoleic acid [34]. The results show (Table 4) that the summer meat composition was particularly high in α-linolenic acid—twice more than the concentrations of the winter meat samples. Supposedly, this could indicate a change in diet from the consumption of predominantly grasses to fewer grasses and more grains. Although the summer meat samples also contained the highest linoleic acid concentrations, it only differed significantly to that of the winter LTL meat (25% vs. 22%). Another explanation could be that the alternative diet (grain- or maize-based) in winter, increased the deposition of saturated fatty acids in the meat and as a result, the winter meat of the springhare had a higher SFA content (Table 4). Furthermore, since it is suggested that both linoleic and α-linolenic acid are essential for human health, as well as the nutritionally influential very long-chain fatty acids, mainly C20:5*n*-3 (eicosapentaenoic acid), and C22:6*n*-3 (docosahexaenoic acid) [35], it is indicated from the findings of this study that the meat of springhare harvested during summer may be more nutritious than meat from the winter. As the foraging behaviours of the springhare used in this study were not determined, only assumptions can be made about the diet of the animals. However, it would be of importance to confirm the effect of diet on the fatty acid content of the meat in future studies.

#### 3.2.4. Effect of Sex on the Fatty Acid Composition

The results for the fatty acid composition (% total FAME) of the different springhare muscles based on sex are shown in Table 5. The Kruskal-Wallis test was used to determine if the means were significant. Apart from two fatty acids (i.e., C17:1 and C22:6*n*3), no other significant differences were found for the fatty acid composition of the male and female meat samples. Fatty acid C17:1 indicated a difference (*p* < 0.05) due to muscle type, being lowest in the LTL meat and similar to the results by North et al. [27]. Fatty acids can vary between muscles of the same sex as each muscle has a distinct anatomical location and function. However, for this study the differences are marginal and more research, especially a larger sample set, is needed to validate the variation in the fatty acid composition of springhare muscles.

Docosahexaenoic acid (DHA) (C22:6*n*3) was higher (*p* < 0.05) in the HL meat of male animals (4.09 ± 0.373%) compared to the LTL (2.89 ± 0.198%) and HL (3.06 ± 0.258%) meat of females. This can be considered a relatively high content as game meat is reported to have DHA concentrations ranging from 0.37 to 2.50% [3], while pork, beef, veal, and chicken range from 0.07 to 1.01% [6], rabbit from 0.15–0.34% [26]. DHA is an essential structural component of the human central nervous system and is required for normal brain function [36]. In humans, DHA is either obtained from the diet (mainly marine origin) or it may be converted in small amounts from eicosapentaenoic acid (EPA, 20:5*n*3). The current results show that springhare meat (particularly from the males) is a source for DHA, where 100 g meat can provide 50 mg DHA in the diet (data not shown). This can contribute to meeting the recommended dietary requirements set for adult males and non-pregnant/non-lactating adult females of 250 mg of DHA and EPA per day [35].

Other noteworthy differences, although not significant, were between the means of the female LTL meat and the male HL meat (Table 5). The female LTL meat had a higher mean value for C16:0 (21%) and *n*-6:*n*-3 (8.4), compared to the male HL meat (18% and 6.3, respectively). Conversely, the male meat had higher means for *n*-3 (6.7) and PUFA:SFA (1.2) than the female LTL meat (5.1 and 1.1, respectively). Lazzaroni et al. [37] found that the effect of sex significantly influenced (*p* < 0.01) the MUFA and PUFA compositions of rabbit meat as a higher content of PUFA and a consequent decrease in MUFA were found in males. These current results indicate that the male springhare HL meat had a slightly more favourable *n*-6:*n*-3 ratio due to its higher *n*-3 content. However, the values for springhare are too low to make any assumption about the biological significance of these differences in ratios. Yet, it could be beneficial for conservation strategies where female numbers need to be sustained by providing evidence that the male meat composition is at least not inferior to that of the female meat, as sexually mature (at approximately 1034 days, 2.5 kg body weight) springhare females only give birth to one young after a gestation period of about 77 days. They thus have a slow reproductive rate for a rodent, and may only reproduce up to three times per year [11].

## 4. Conclusions

The findings of this study reveal that the springhare is a viable meat source with a high protein content and favorable low total lipid content. It also has a favorable fatty acid composition, where animals sourced during summer have a higher content of unsaturated fatty acids, this is particularly evident for the meat of the hind leg. It is recommended to implement a system where the meat is valorized and distributed to populations, especially in cases where springhare are hunted as pests. The results also reveal limited significant differences in the overall chemical composition of the meat from male and female springhare, except for the noteworthy higher (*p* < 0.05) DHA (C22:6*n*3) concentration and *n*-6:*n*-3 ratio in the HL meat of male animals. However, additional research is still needed to validate the findings of the current study and further determine the meat quality of springhare, focusing on aspects such as its organoleptic quality, sensory and physical characteristics, enzymatic properties, and microbial quality.

## Figures and Tables

**Table 1:** The mean ± standard error values for the carcass characteristics of the springhare based on season and sex.

	Season			Sex		
Carcass Characteristics	Summer (*n* = 10)	Winter (*n* = 10)	*p*	Male (*n* = 10)	Female (*n* = 10)	*p*
Dead weight (kg)	2.4 ± 0.2	2.6 ± 0.1	0.453	2.6 ± 0.1	2.4 ± 0.1	0.227
Carcass weight (kg)	1.6 ± 0.1	1.6 ± 0.0	0.821	1.6 ± 0.1	1.5 ± 0.1	0.251
Dress out percentage ^1^ (%)	64.3 ± 0.7	62.1 ± 0.7	0.039	63.0 ± 0.9	63.5 ± 0.6	0.691
Total weight ^2^ (g)	1450.3 ± 92.3	1485.8 ± 46.0	0.735	1514.3 ± 74.6	1421.9 ± 68.6	0.374
Forearms (both, g)	92.6 ± 5.3	97.2 ± 3.3	0.476	98.4 ± 4.1	91.4 ± 4.5	0.261
Loins ^3^ (meat, g)	206.7 ± 18.2	215.2 ± 12.5	0.703	226.8 ± 18.5	195.1 ± 10.0	0.148
Hind legs (intact, g)	646.1 ± 32.9	617.4 ± 24.2	0.492	649.8 ± 31.3	613.7 ± 25.8	0.385
Hind legs (bones, g)	123.3 ± 7.1	113.0 ± 7.2	0.319	116.7 ± 8.9	119.7 ± 5.2	0.776
Hind legs (meat, g)	519.0 ± 29.6	475.3 ± 24.9	0.272	502.9 ± 33.2	491.4 ± 22.0	0.777
Tail (g)	70.9 ± 3.6	61.1 ± 1.8	0.026	67.1 ± 3.7	65.0 ± 2.7	0.653
Rest of carcass ^4^ (g)	428.5 ± 43.8	492.9 ± 25.3	0.219	468.1 ± 34.0	453.2 ± 40.0	0.780

(*n*) Number of animals; ^1^ Dress out percentage is the carcass weight divided by the dead weight times 100; ^2^ Total weight is the weight of the defrosted body, without skin, head, feet and intestines; ^3^ Loins are the right and left *Longissimus thoracis et lumborum* (LTL) muscles; ^4^ Rest of carcass is without the arms, loins, legs and tails; Means in the same corresponding row (season or sex) with *p* < 0.05 are significantly different according to Fisher’s Least significant difference.

**Table 2:** The mean ± standard error values for the proximate composition (g/100 g meat) of the different springhare muscles (loin and hind leg) based on season and sex.

	Season			Sex		
Parameters	Summer (*n* = 10)	Winter (*n* = 10)	*p*	Male (*n* = 10)	Female (*n* = 10)	*p*
Loin muscles ^1^						
Moisture	75.1 ± 0.3	75.7 ± 0.3	0.140	75.4 ± 0.3	75.4 ± 0.3	0.991
Protein	22.7 ± 0.3	22.8 ± 0.3	0.855	22.7 ± 0.2	22.8 ± 0.4	0.867
Total lipid	1.3 ± 0.1	1.2 ± 0.1	0.611	1.3 ± 0.1	1.2 ± 0.1	0.683
Ash	1.0± 0.0	1.1 ± 0.0	0.002	1.0 ± 0.0	1.0 ± 0.0	0.933
Hind leg muscles						
Moisture	75.5 ± 0.1	76.0 ± 0.2	0.038	75.6 ± 0.2	75.9 ± 0.2	0.237
Protein	22.2 ± 0.3	22.5 ± 0.2	0.372	22.5 ± 0.2	22.2 ± 0.3	0.277
Total lipid	1.3 ± 0.1	1.1 ± 0.1	0.108	1.2 ± 0.1	1.2 ± 0.1	0.665
Ash	0.9 ± 0.0	1.1 ± 0.0	0.005	1.0 ± 0.0	1.0 ± 0.0	0.183

(*n*) Number of animals; ^1^ Loins are the right and left *Longissimus thoracis et lumborum* (LTL) muscles; Means in the same corresponding row (season or sex) with *p* < 0.05 are significantly different according to Fisher’s Least significant difference.

**Table 3:** The mean ± standard error values for the fatty acid composition (% total FAME) of the springhare meat.

Fatty Acids	Name	Mean	Standard Error
C13:0	Tridecylic acid	0.04	0.005
C14:0	Myristic acid	0.27	0.037
C15:0	Pentadecylic acid	0.22	0.011
C16:0	Palmitic acid	19.45	0.454
C18:0	Stearic acid	19.53	0.348
C20:0	Arachidic acid	0.07	0.003
C21:0	Heneicosylic acid	0.33	0.023
C22:0	Behenic acid	0.12	0.009
C24:0	Lignoceric acid	0.21	0.014
C16:1	Palmitoleic acid	0.30	0.025
C17:1	Heptadecenoic acid	0.20	0.021
C18:1*n*9c	Oleic acid	12.69	0.765
C20:1	Gondoic acid	0.17	0.018
C22:1*n*9	Erucic acid	0.14	0.013
C18:2*n*6c	Linoleic acid	23.72	0.650
C18:3*n*6	γ-Linolenic acid	0.25	0.021
C18:3*n*3	α-Linolenic acid	1.52	0.186
C20:2*n*6	Eicosadienoic acid	0.05	0.004
C20:3*n*6	Dihomo-γ-linolenic acid	1.10	0.039
C20:3*n*3	Eicosatrienoic acid	0.26	0.017
C20:4*n*6	Arachidonic acid	15.14	0.632
C20:5*n*3	Eicosapentaenoic acid	0.62	0.050
C22:6*n*3	Docosahexaenoic acid	3.47	0.178
ΣSFA	Total saturated fatty acids	40.33	0.560
ΣMUFA	Total mono-unsaturated fatty acids	13.55	0.791
ΣPUFA	Total polyunsaturated fatty acids	46.12	0.623
PUFA:SFA	Polyunsaturated fatty acid:Saturated fatty acid	1.15	0.024
Σ*n*-6	Total *n*-6 PUFA	40.25	0.561
Σ*n*-3	Total *n*-3 PUFA	5.87	0.250
(*n*-6):(*n*-3)	Total omega 6:Total omega 3	7.46	0.429

Non-detected fatty acids: C6:0, C8:0, C10:0, C11:0, C12:0, C23:0, C14:1, C15:1, C18:1*n*9t, C24:1, C18:2*n*6t, C22:2*n*6; Number of animals used: 20; Muscles combined and analysed per animal: the left and right *Longissimus thoracis* et *lumborum* muscles (from the intermediate part) and all the hindleg muscles (from the hind part).

**Table 4:** The mean ± standard error values for the fatty acid composition (% total FAME) of the different summer and winter springhare muscles.

	Loin Muscles (*n* = 20) ^1^		Hind Leg Muscles (*n* = 20)		
	Season		Season		*p*
Fatty Acids	Summer	Winter	Summer	Winter	
C13:0	0.06 ^a^ ± 0.008	0.03 ^b^ ± 0.010	0.03 ^b^ ± 0.010	0.06 ^a^ ± 0.007	0.012
C14:0	0.37 ± 0.096	0.23 ± 0.037	0.23 ± 0.094	0.23 ± 0.053	0.158
C15:0	0.24 ± 0.017	0.23 ± 0.016	0.18 ± 0.026	0.23 ± 0.024	0.195
C16:0	20.71 ^a^ ± 0.705	19.74 ^ab^ ± 0.743	17.21 ^b^ ± 1.072	20.14 ^ab^ ± 0.760	0.043
C18:0	19.13 ± 0.759	19.15 ± 0.555	19.88 ± 0.977	19.97 ± 0.440	0.712
C20:0	0.06 ^b^ ± 0.005	0.06 ^b^ ± 0.004	0.09 ^a^ ± 0.007	0.05 ^b^ ± 0.004	<0.001
C21:0	0.38 ± 0.060	0.30 ± 0.021	0.39 ± 0.058	0.28 ± 0.019	0.541
C22:0	0.09 ^b^ ± 0.010	0.12 ^ab^ ± 0.015	0.15 ^a^ ± 0.027	0.13 ^a^ ± 0.012	0.049
C24:0	0.20 ^ab^ ± 0.029	0.17 ^b^ ± 0.012	0.19 _ab_ ± 0.026	0.29 ^a^ ± 0.025	0.009
C16:1	0.26 ± 0.055	0.30 ± 0.039	0.30 ± 0.064	0.33 ± 0.044	0.685
C17:1	0.12 ^b^ ± 0.024	0.14 ^b^ ± 0.022	0.36 ^a^ ± 0.048	0.17 ^ab^ ± 0.012	<0.001
C18:1*n*9c	11.40 ± 1.593	14.61 ± 1.981	12.07 ± 1.424	12.68 ± 1.001	0.656
C20:1	0.15 ± 0.024	0.19 ± 0.030	0.22 ± 0.057	0.14 ± 0.016	0.557
C22:1*n*9	0.14 ± 0.016	0.17 ± 0.038	0.11 ± 0.027	0.13 ± 0.016	0.426
C18:2*n*6c	25.16 ± 1.495	21.71 ± 0.833	25.41 ± 1.672	22.59 ± 0.671	0.121
C18:3*n*6	0.19 ^b^ ± 0.025	0.26 ^ab^ ± 0.031	0.38 ^a^ ± 0.053	0.17 ^b^ ± 0.015	0.005
C18:3*n*3	1.82 ^ab^ ± 0.397	0.95 ^b^ ± 0.157	2.30 ^a^ ± 0.512	1.01 ^b^ ± 0.114	0.026
C20:2*n*6	0.04 ^ab^ ± 0.004	0.05 ^ab^ ± 0.004	0.07 ^a^ ± 0.010	0.03 ^b^ ± 0.005	0.018
C20:3*n*6	1.14 ± 0.092	1.12 ± 0.068	1.09 ± 0.095	1.03 ± 0.055	0.780
C20:3*n*3	0.29 ^ab^ ± 0.030	0.17 ^b^ ± 0.019	0.34 ^a^ ± 0.042	0.23 ^ab^ ± 0.021	0.007
C20:4*n*6	14.12 ± 1.354	16.15 ± 1.280	14.36 ± 1.603	15.93 ± 0.713	0.665
C20:5*n*3	0.67 ± 0.132	0.58 ± 0.073	0.66 ± 0.120	0.56 ± 0.072	0.853
C22:6*n*3	3.23 ± 0.380	3.50 ± 0.366	3.63 ± 0.450	3.52 ± 0.246	0.876
ΣSFA	41.27 ± 1.267	40.10 ± 1.127	38.48 ± 1.106	41.47 ± 0.842	0.339
ΣMUFA	12.07 ± 1.674	15.40 ± 2.051	13.27 ± 1.446	13.45 ± 1.058	0.670
ΣPUFA	46.66 ± 0.868	44.49 ± 1.430	48.25 ± 1.488	45.07 ± 0.872	0.084
PUFA:SFA	1.14 ^ab^ ± 0.035	1.11 ^ab^ ± 0.040	1.27 ^a^ ± 0.062	1.09 ^b^ ± 0.035	0.044
Σ*n*-6	40.65 ± 0.940	39.29 ± 1.251	41.32 ± 1.405	39.75 ± 0.869	0.408
Σ*n*-3	6.02 ^ab^ ± 0.549	5.20 ^b^ ± 0.341	6.93 ^a^ ± 0.629	5.32 ^b^ ± 0.241	0.049
(*n*-6):(*n*-3)	7.60 ± 1.089	7.76 ± 0.423	6.87 ± 1.268	7.62 ± 0.393	0.077

(*n*) Number of animals; ^1^ Loins are the right and left *Longissimus thoracis et lumborum* (LTL) muscles; (ΣSFA) Total saturated fatty acids; (ΣMUFA) Total mono-unsaturated fatty acids; (ΣPUFA) Total polyunsaturated fatty acids; (PUFA:SFA) Polyunsaturated fatty acid:Saturated fatty acid ratio; (Σ*n*-6) Total *n*-6 PUFA; (Σn-3) Total *n*-3 PUFA; (*n*-6:*n*-3) Total omega 6:Total omega 3 ratio; ^a-b^ Means in the same row with *p* < 0.05 are significantly different according to Dunn’s post-hoc test; Non-detected fatty acids: C6:0, C8:0, C10:0, C11:0, C12:0, C23:0, C14:1, C15:1, C18:1*n*9t, C24:1, C18:2*n*6t, C22:2*n*6.

**Table 5:** The mean ± standard error values for the fatty acid composition (% total FAME) of the different male and female springhare muscles.

	Loin Muscles (*n* = 20) ^1^		Hind Leg Muscles (*n* = 20)		
	Sex		Sex		*p*
Fatty Acids	Male	Female	Male	Female	
C13:0	0.04 ± 0.008	0.06 ± 0.011	0.04 ± 0.010	0.04 ± 0.010	0.737
C14:0	0.32 ± 0.100	0.29 ± 0.039	0.17 ± 0.062	0.28 ± 0.084	0.129
C15:0	0.23 ± 0.013	0.24 ± 0.019	0.19 ± 0.025	0.21 ± 0.028	0.386
C16:0	19.79 ± 0.471	20.66 ± 0.914	17.98 ± 0.863	19.37 ± 1.162	0.243
C18:0	18.97 ± 0.646	19.32 ± 0.677	19.50 ± 0.660	20.35 ± 0.820	0.551
C20:0	0.06 ± 0.004	0.06 ± 0.004	0.07 ± 0.006	0.07 ± 0.010	0.495
C21:0	0.33 ± 0.034	0.34 ± 0.057	0.34 ± 0.035	0.32 ± 0.057	0.717
C22:0	0.11 ± 0.015	0.10 ± 0.012	0.13 ± 0.009	0.15 ± 0.028	0.108
C24:0	0.17 ± 0.017	0.20 ± 0.026	0.23 ± 0.028	0.25 ± 0.032	0.109
C16:1	0.30 ± 0.046	0.27 ± 0.049	0.30 ± 0.034	0.33 ± 0.070	0.813
C17:1	0.13 ^b^ ± 0.023	0.13 ^b^ ± 0.023	0.26 ^a^ ± 0.039	0.26 ^ab^ ± 0.054	0.004
C18:1*n*9c	12.76 ± 1.570	13.26 ± 2.135	12.92 ± 1.244	11.84 ± 1.199	0.969
C20:1	0.16 ± 0.022	0.17 ± 0.032	0.21 ± 0.057	0.15 ± 0.020	0.900
C22:1*n*9	0.17 ± 0.037	0.14 ± 0.018	0.14 ± 0.026	0.10 ± 0.015	0.345
C18:2*n*6c	22.96 ± 1.063	23.92 ± 1.552	23.93 ± 0.816	24.07 ± 1.738	0.897
C18:3*n*6	0.24 ± 0.035	0.21 ± 0.025	0.29 ± 0.052	0.26 ± 0.054	0.891
C18:3*n*3	1.47 ± 0.399	1.31 ± 0.252	1.84 ± 0.529	1.47 ± 0.284	0.872
C20:2*n*6	0.05 ± 0.004	0.04 ± 0.004	0.05 ± 0.010	0.05 ± 0.010	0.912
C20:3*n*6	1.12 ± 0.061	1.14 ± 0.096	1.07 ± 0.062	1.05 ± 0.092	0.765
C20:3*n*3	0.23 ± 0.018	0.23 ± 0.041	0.30 ± 0.031	0.27 ± 0.043	0.344
C20:4*n*6	16.01 ± 1.428	14.26 ± 1.220	15.28 ± 0.970	15.02 ± 1.507	0.772
C20:5*n*3	0.56 ± 0.063	0.70 ± 0.135	0.50 ± 0.050	0.72 ± 0.122	0.497
C22:6*n*3	3.84 ^ab^ ± 0.440	2.89 ^b^ ± 0.198	4.09 ^a^ ± 0.373	3.06 ^b^ ± 0.258	0.039
ΣSFA	40.02 ± 0.880	41.35 ± 1.441	38.78 ± 0.938	41.17 ± 1.110	0.375
ΣMUFA	13.51 ± 1.634	13.96 ± 2.224	13.88 ± 1.274	12.84 ± 1.237	0.946
ΣPUFA	46.47 ± 1.112	44.69 ± 1.284	47.34 ± 1.089	45.98 ± 1.498	0.474
PUFA:SFA	1.16 ± 0.031	1.09 ± 0.040	1.23 ± 0.049	1.13 ± 0.062	0.259
Σ*n*-6	40.38 ± 0.975	39.56 ± 1.249	40.61 ± 0.957	40.46 ± 1.396	0.887
Σ*n*-3	6.09 ± 0.451	5.12 ± 0.446	6.73 ± 0.489	5.52 ± 0.526	0.142
(*n*-6):(*n*-3)	6.99 ± 0.567	8.37 ± 0.969	6.31 ± 0.452	8.18 ± 1.182	0.283

(*n*) Number of animals; ^1^ Loins are the right and left *Longissimus thoracis et lumborum* (LTL) muscles; (ΣSFA) Total saturated fatty acids; (ΣMUFA) Total mono-unsaturated fatty acids; (ΣPUFA) Total polyunsaturated fatty acids; (PUFA:SFA) Polyunsaturated fatty acid:Saturated fatty acid ratio; (Σ*n*-6) Total *n*-6 PUFA; (Σ*n*-3) Total *n*-3 PUFA; (*n*-6:*n*-3) Total omega 6:Total omega 3 ratio; ^a-b^ Means in the same row with *p* < 0.05 are significantly different according to Dunn’s post-hoc test; Non-detected fatty acids: C6:0, C8:0, C10:0, C11:0, C12:0, C23:0, C14:1, C15:1, C18:1*n*9t, C24:1, C18:2*n*6t, C22:2*n*6.
